# Acupuncturation of the Arteries

**Published:** 1831

**Authors:** 


11. *Acupuncturation of the Arteries.*—At a late meeting of the Parisian Academy of Sciences, M. Velpeau suggested the acupuncture of arteries as an advantageous substitute for ligature. —*Ib.*

